# Supplementary material for: NoxO1 Knockout Promotes Longevity in Mice
Source: Antioxidants (Basel). 2020 Mar 10;9(3):226. doi: 10.3390/antiox9030226 (PMC7139303; doi:10.3390/antiox9030226)
Supplement: Supplementary file 1 [file antioxidants-09-00226-s001.pdf]

A Body weight at 2 month of age

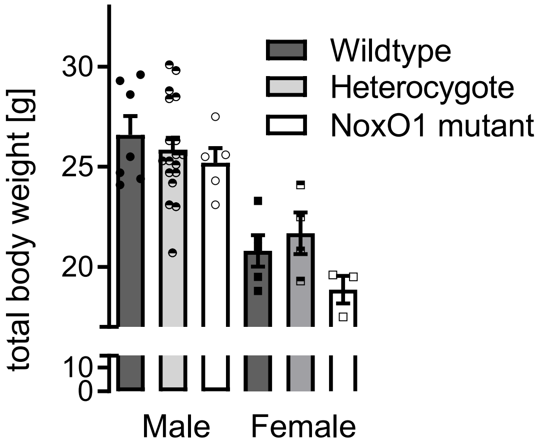

B AmplexRed assay from colon tissue

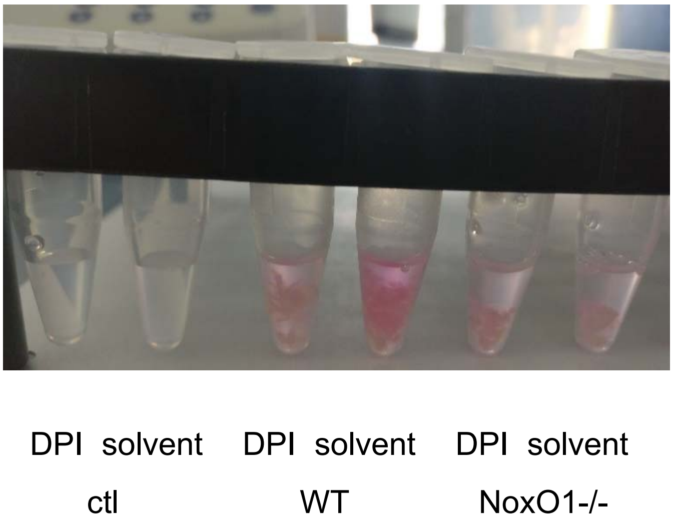

C AmplexRed assay normalized to tissue weight

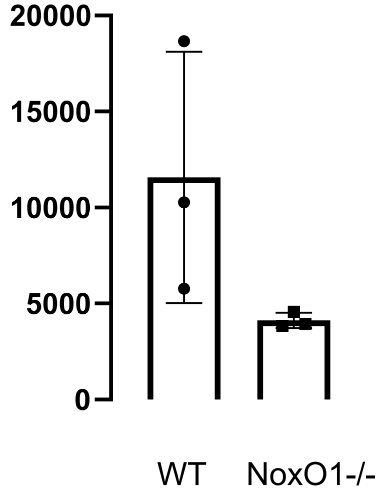

Supplemental figure 1
